# Supplementary material for: GP IIb/IIIa Receptor Inhibitors in Mechanically Ventilated Patients with Cardiogenic Shock due to Myocardial Infarction in the Era of Potent P2Y12 Receptor Antagonists
Source: J Clin Med. 2022 Dec 14;11(24):7426. doi: 10.3390/jcm11247426 (PMC9783576; doi:10.3390/jcm11247426)
Supplement: Supplementary file 1 [file jcm-11-07426-s001.zip › jcm-2040751-supplementary.pdf]

Supplementary Table S1. Hospital complications and CPC score.

Variable	NO – GPI<br>N = 75	GPI<br>N = 78	POPULATION<br>N = 153	p
Bronchopneumonia	48 (64.0)	49 (63.1)	97 (63.8)	1.00
Renal replacement therapy	7(9.3)	8 (10.3)	15 (9.8)	1.00
Multiorgan failure	18 (24.0)	18 (23.1)	36 (23.5)	1.00
Sepsis	20 (26.7)	21 (26.9)	41 (26.8)	1.00
Thromboembolic events	2 (2.7)	1 (1.3)	3 (2.09	0.61
Coma or vegetative state	16 (21.3)	2 (2.6)	18 (11.8)	<0.0001
Acute kidney injury	33 (56.9)	37 (50.0)	70 (53.0)	0.48
Intracranial hemorrhage	3 (4.0)	3 (3.8)	6 (3.9)	1.00

CPR = cardiopulmonary resuscitation; GPI= IIb/IIIa receptor inhibitors; P2Y12=P2Y12 receptor antagonists.
